# Applying Spatial Transcriptomics to the Human Heart: A Practical Guide for Translational Studies

**DOI:** 10.1016/j.jacbts.2026.101730

**Published:** 2026-09-22

**Authors:** Hongyan Tao, James Cranley, Farid Kadyrov, Haewon Shin, Raphaël Blain, Jagdeep Nanchahal, Adrien Hallou, Kaiyue Zhang, Maarten Hulsmans, Andrew JM. Lewis

**Affiliations:** a Division of Cardiovascular Medicine, Radcliffe Department of Medicine, University of Oxford, Oxford, United Kingdom; b Department of Medicine, University of Cambridge, Cambridge, United Kingdom; c Center for Systems Biology, Massachusetts General Brigham and Harvard Medical School, Boston, Massachusetts, USA; d Department of Radiology, Massachusetts General Brigham and Harvard Medical School, Boston, Massachusetts, USA; e Center for Cardiovascular Research, Division of Cardiology, Department of Medicine, Washington University School of Medicine, Saint Louis, Missouri, USA; f Kennedy Institute of Rheumatology, University of Oxford, Oxford, United Kingdom; g Department of Biomedical Engineering, Columbia University, New York, New York, USA

**Keywords:** cardiomyocyte segmentation, cardiovascular magnetic resonance imaging, deconvolution, infarct border zone, multiomic integration, spatial transcriptomics

## Abstract

Spatial transcriptomics and single-cell methods are changing the questions we can ask about the human cardiovascular system. In this review, we share lessons learned from applying spatial profiling to human cardiovascular samples. We walk through experimental considerations, sampling, fixation, and ribonucleic acid quality control, then compare sequencing-based and imaging-based platforms within a 2-stage "discovery then validation" framework. We propose a heart-aware analytical pipeline: spatially informed quality control, normalization respecting the outsized transcriptome of cardiomyocytes, thoughtful segmentation, and honest assessment of reproducibility. We consider how spatial data can be woven together with single-cell, epigenomic, and proteomic layers, and look ahead to emerging frontiers including integration with cardiac imaging foundation models. Our hope is that spatially resolved molecular data will reveal new tissue niches to target for precision cardiovascular therapies.

## Highlights

• Spatial context exposes disease programs across infarct, border and remote zones.
• Practical guidance on tissue handling, platform choice, and heart-aware analysis.
• Orthogonal assays and multiomic integration prioritize therapeutic targets.
• Coregistration with cardiac imaging links tissue biology to in vivo phenotypes.

"Well, now that we have seen each other," said the Unicorn, "if you'll believe in me, I'll believe in you.”

—Lewis Carroll, Alice's Adventures in Wonderland.

Cardiovascular disease is inherently spatial: the heart itself is structurally asymmetric across chambers, walls, and transmural layers, and disease manifests in spatially distinct locations: atherosclerosis emerges at vessel branching points, infarcts expand wavefront-like from endocardium to epicardium, and arrhythmias emanate from sites of disrupted tissue architecture. Understanding how cells and genes interact within the spatial context of their tissue architecture may be the key to unraveling new mechanisms and therapies.^1^ Yet as powerful as they are, traditional bulk or dissociated single-cell assays sacrifice this spatial context,^2^^,^^3^ and recovering it requires tools that couple spatially resolved molecular data with advanced computation.^4^, ^5^, ^6^, ^7^ The ambition to map gene expression to tissue coordinates is not new,^8^ and various forms of in situ hybridization have long been used to visualize specific gene transcripts in their native spatial context.^9^, ^10^, ^11^ The modern era of unbiased, high-throughput spatial transcriptomics (ST) was launched by Ståhl et al,^12^ who showed that transcriptomes could be captured from intact tissue sections using arrays of positionally barcoded primers, first validated in mouse brain and human breast cancer. This foundational work established that gene expression could be quantified while preserving its precise position within a section, opening the door to entirely new bioinformatics analyses. *Nature Methods* named spatially resolved transcriptomics as Method of the Year in 2021.^13^ This designation reflected a period of rapid expansion in the field, as diverse new platforms emerged.^14^, ^15^, ^16^, ^17^, ^18^, ^19^, ^20^, ^21^, ^22^

Several developments make this a timely moment to review spatial technologies in the human heart. First, robust protocols for formalin-fixed paraffin-embedded (FFPE) tissue have opened access to archival samples, enabling retrospective studies that link spatial molecular patterns to long-term patient trajectories.^23^, ^24^, ^25^, ^26^ Second, platform capabilities have expanded, narrowing the traditional trade-off between spatial resolution and transcriptome coverage. Imaging platforms can now achieve unbiased, whole-transcriptome profiling at subcellular resolution even in complex primary tissue.^27^ Third, computational approaches have matured to handle the unique properties of cardiac tissue, where large, multinucleated cardiomyocytes challenge standard pipelines. Computational tools can now leverage histology to delineate anatomical zones and use probabilistic deconvolution to resolve cellular composition within mixed signals, although most require adaptation rather than being purpose-built for cell types predominant in heart tissue.^28^, ^29^, ^30^, ^31^ Recent reviews have surveyed spatial transcriptomic platforms and their applications in cardiac tissue.^32^^,^^33^ Here, we focus particularly on practical experimental decisions, failure modes, and postprocessing and informatics technologies for human cardiac ST (Central Illustration).

ST platforms broadly divide into 2 categories: sequencing-based ST (SBST, eg, Visium, Visium HD, Stereo-seq) captures the whole transcriptome across large tissue areas but at lower spatial resolution, whereas imaging-based ST (IBST, eg, Xenium, CosMx, MERFISH) achieves single-molecule detection at subcellular resolution but is limited to targeted gene panels. Both approaches present distinct challenges in cardiac tissue, which we address in turn.

## Samples: Overcoming the Biophysical Barriers of Myocardium

Cardiac tissue is technically demanding to work with: Large myocytes, anisotropic fibre bundles, dense matrix, and high autofluorescence each pose distinct challenges for ST.

### The challenge of cardiac tissue architecture

The primary challenge is the cardiomyocyte itself. Unlike fetal cardiomyocytes, these cells are large (100-150 μm length), irregular in shape, and often multinucleated.^34^^,^^35^ This morphology creates important issues depending on the platform used. In first-generation SBST, each 55 μm capture spot encompasses multiple cells, mixing cardiomyocyte signal with that of smaller interstitial cells and requiring computational deconvolution. In second-generation high-resolution SBST (eg, Visium HD), the situation inverts: each cardiomyocyte spans multiple capture spots, and transcripts must be aggregated back into biologically meaningful cells. In imaging-based ST, standard segmentation algorithms assume roughly circular cells expanding from a single nucleus, since most operate in 2-dimensional. These approaches struggle with elongated, multinucleated myocytes.^35^ Strategies to address segmentation in cardiac tissue are discussed under Cell Segmentation below compared in Section 4.2.

Beyond the individual cell, tissue architecture adds further complexity. Similar to other striated muscle, gene expression in the heart varies significantly based on section orientation relative to the myocardial fibre helix (eg, longitudinal vs transverse) and transmural position.^36^^,^^37^ Furthermore, scar and perivascular regions are paucicellular and dense with matrix components, slowing probe diffusion through the tissue.^38^^,^^39^ Finally, myocardium exhibits autofluorescence from lipofuscin and collagen, generating optical noise that obscures signals in imaging assays.^40^

### Tailored processing workflows

Success depends on adapting the workflow to these specific constraints and the preservation method used. Although fresh frozen (FF) tissue offers higher ribonucleic acid (RNA) quality for unbiased discovery, clinical archives are more likely to contain FFPE blocks. The major challenge with FFPE is RNA degradation: formalin fixation itself introduces strand breaks through formaldehyde-mediated cross-linking, and prolonged archival storage further degrades transcript integrity, compounded by suboptimal storage temperature or humidity.^41^ Beyond baseline degradation, fixation duration matters. Under fixation compromises morphology and allows RNA leakage, whereas over fixation excessively crosslinks transcripts, hindering retrieval without damaging tissue architecture. This trade-off is more problematic for in situ hybridization methods, where RNA must be accessed within intact tissue; sequencing-based platforms image the section before RNA release, partly mitigating the effects of over-fixation. Probe design also matters: platforms using short binding sequences (eg, Xenium, 16 nt) tolerate highly fragmented RNA better than those requiring longer probe binding regions or poly(A) capture, which can fail on degraded archival samples. In infarct border zones, areas of necrosis and immune infiltration are more susceptible to degradation.^42^ In such samples, RNA quality is better assessed using the DV200 metric (percentage of fragments >200 nucleotides; target >30%, although higher values correlate with greater assay sensitivity) rather than the RNA Integrity Number, an algorithm dependent on ribosomal peaks that performs poorly on fragmented samples.^23^

Specific anatomical features also require tailored protocols. Cardiovascular samples often contain calcification, such as in aortic stenosis or atherosclerotic plaques, which may resist standard sectioning techniques. Since standard acid decalcification destroys RNA, modified protocols using controlled ethylenediaminetetraacetic acid-based decalcification at neutral pH are required to preserve transcript integrity in these mineralized niches.^43^^,^^44^ For imaging assays, the autofluorescence of myocardium must be managed. Autofluorescence can be reduced with Sudan Black B (0.1% in 70% ethanol) or lipofuscin-targeting quenchers such as TrueBlack,^40^^,^^45^ and detection in far-red channels, where cardiac autofluorescence is lower, improves signal-to-noise ratio.^46^

Strategic sampling helps mitigate selection bias. Published developmental and anatomical atlases^26^^,^^47^ provide a reference framework for identifying specific structures such as conduction tracts, guiding region of interest (ROI) selection based on established histologic landmarks. To further minimize bias in ROI selection, emerging computational frameworks like S2-omics^48^ can analyse rapid H&E scans to predict regions of maximal transcriptomic yield. Before committing to a full run, each sample should clear basic quality gates: DV200 on adjacent curls, RNA Integrity Number for FF, and acceptable signal-to-noise in a pilot region for imaging assays (Figure 1).

**Figure 1:**
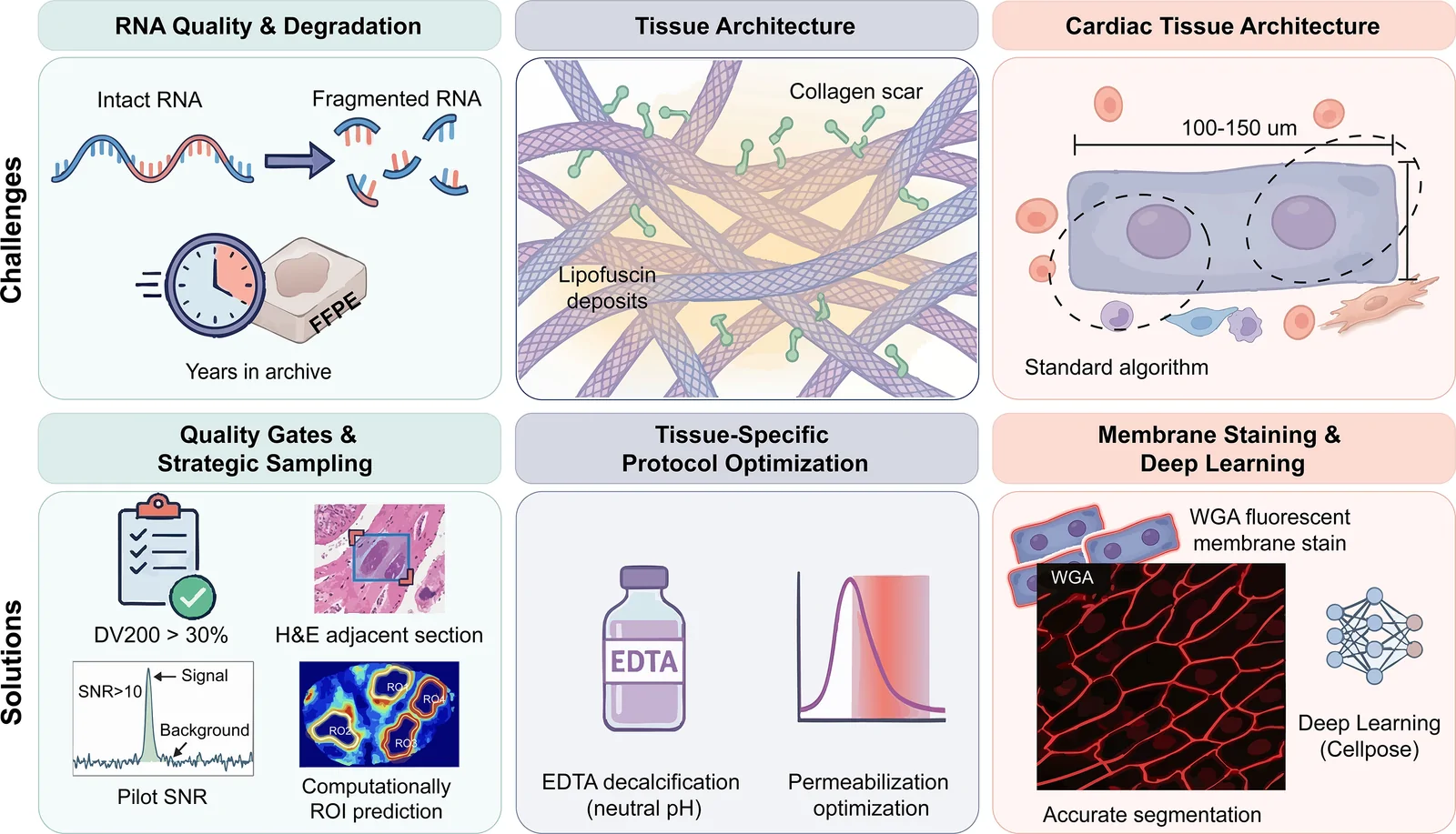
Cardiac Tissue Challenges and Tailored Solutions for Spatial Transcriptomics Top row: 3 principal barriers. Ribonucleic acid (RNA) quality deteriorates with prolonged formalin-fixed paraffin-embedded (FFPE) archival storage and is accelerated by necrosis and immune infiltration in infarct border zones. Dense collagen scars and lipofuscin deposits impede probe diffusion and generate autofluorescence. Adult cardiomyocytes (100-150 μm, often binucleated) defeat standard segmentation algorithms that assume roughly spherical cells. Corresponding solutions (bottom row): samples should pass 4 quality checkpoints before committing to a full run—RNA fragment quality (DV200 > 30%), section orientation confirmed by adjacent hematoxylin and eosin (H&E), acceptable signal-to-noise in a pilot imaging region, and computational region of interest (ROI) prediction to direct acquisition toward regions of maximal transcriptomic yield. Tissue-specific protocol optimization includes ethylenediaminetetraacetic acid (EDTA)-based decalcification at neutral potential of hydrogen (pH), autofluorescence quenching (Sudan Black B or TrueBlack), and adjusted permeabilization times for fibrotic regions. Wheat germ agglutinin (WGA) membrane staining supplies the boundary information that nuclear-only inputs lack, improving cardiomyocyte segmentation for downstream transcript assignment (Figure 2). SNR = signal-to-noise ratio; um = micrometers.

## Platforms and Workflows for Spatial Transcriptomics in the Human Heart

For human myocardium, the choice between sequencing-based and imaging-based platforms has historically hinged on a trade-off between spatial resolution and transcriptome breadth. However, a recent practical comparison of 7 commercial platforms^49^^,^^50^ emphasizes that as resolutions converge, researchers must now weigh gene coverage against detection sensitivity more carefully. Sequencing-based methods often remain better suited to discovering novel isoforms or rare cell states, whereas imaging platforms excel at subcellular localization of targeted panels in complex cardiac tissue (Table 1).

**Table 1:** Comparison of Major Spatial Platforms for Human Heart Tissue

Feature	Curio (Slide-Seq V2)	10x Visium/Visium HD	10x Xenium	10x Atera	NanoString CosMx SMI	Vizgen MERFISH
Primary utility	Discovery (total RNA)	Unbiased discovery	Targeted validation	Discovery at scale	Discovery and validation	Targeted validation
modality	Sequencing-based	Sequencing-based	Imaging-based	Imaging-based	Imaging-based	Imaging-based
Resolution	approximately 10 μm (bead)	55 μm/approximately 2 μm (HD)	Subcellular	Subcellular	Subcellular	Subcellular
Analytes	Total RNA (poly(dT) capture)	mRNA (probe-based); or poly(A) RT in HD 3′	mRNA (probe-based)	mRNA (probe-based)	mRNA (probe-based)	mRNA (probe-based)
Plexity	WTx	Approximately 18,000-19,000 (probe panel); WTx (HD 3′)	Approximately 5,000 (5K panel); custom and add-on panels supported	Approximately 18,000; optional 1,000-gene panel stacking	Approximately 1,000-6,000; approximately 18,000	Approximately 500-1,000
FFPE compatible	FF only	Excellent (probe panel); FF only (HD 3′)	Excellent	Yes (FF and FFPE)	Excellent	Good
Heart specifics	Very sparse (approximately 100 UMIs/bead), high mito/ribo contamination, no H&E	Robust; HD requires nuclei-aware binning	Fast; requires segmentation tuning for cardiomyocytes	No cardiac data published to date	High sensitivity; WTx mode is data-heavy	High sensitivity; watch autofluorescence
Best for	Niche cases needing unbiased total RNA; limited cardiac utility to date	Mapping infarct gradients; transcriptome-wide discovery	High-throughput screening; custom cardiac panels	Large-cohort WTx studies	Deep profiling of rare cell states and niches	Mapping spatial organization of known genes

FF = fresh frozen; FFPE = formalin-fixed paraffin-embedded; H&E = hematoxylin and eosin; HD = high definition; SMI = Spatial Molecular Imaging; UMI = unique molecular identifier; WTx = whole transcriptome.

### Sequencing-based capture: Unbiased discovery

The newer Visium HD overcomes the resolution limitations of the first-generation Visium (55 μm spots, each encompassing multiple cells) with high-density bins (2-micron fundamental resolution, output at 8 microns), enabling cellular or, for some cell types, subcellular resolution while retaining slide-scale coverage. Visium HD exists in 2 configurations. The probe-based panel targets approximately 18,000 to 19,000 protein-coding genes (typically 2-3 probes per gene) and is compatible with FFPE, FF, and fixed frozen tissue. The separate Visium HD 3′ assay uses poly(A) reverse transcription on FF tissue only, providing genuinely unbiased discovery including novel transcripts. It is particularly powerful for defining sharp domain boundaries along infarct edges, though it requires heavier computational resources to aggregate bins into biologically meaningful cells, for example using bin2cell^51^ or nuclei-aware segmentation pipelines.^50^

Emerging academic-origin platforms like Stereo-seq offer even finer spatial resolution,^14^ and have recently advanced to “Stereo-seq V2.”^52^^,^^53^ Unlike standard poly(A) capture, this method employs random primers to capture total RNA. This enables profiling of total RNA, including noncoding transcripts that regulate hypertrophic signaling, and even bacterial RNA, potentially relevant in infective endocarditis, at subcellular resolution in FFPE tissue. For cardiovascular applications, where regulatory noncoding RNAs and microbiome interactions are increasingly recognized, this broader capture may prove valuable.

### In situ imaging: High-resolution validation

Imaging platforms including Xenium, CosMx, and MERFISH offer single-molecule subcellular resolution, making them well suited to validating specific micro-niches such as perivascular fibrosis or conduction system regions. All perform well on FFPE tissue, and recent advances have expanded their capabilities considerably: CosMx SMI (NanoString)^27^ now offers whole-transcriptome profiling (approximately 18,000 genes), blurring the traditional boundary between sequencing-based discovery and imaging-based validation.

Yet imaging in adult myocardium poses challenges regardless of platform. Standard nuclear expansion-based segmentation often misassigns transcripts when cells are large, irregular, or binucleated.^54^ Advanced computational solutions are therefore required, whether image-based (Cellpose^28^), transcript-based (Baysor^55^), or membrane-guided; these are compared below (Figure 2).

The choice between platforms often comes down to study design and resources. Targeted panels (typically 200-1,000 genes) offer faster runs, manageable data volumes, and sufficient depth for hypothesis-driven validation, as demonstrated in a recent atlas of the developing heart, which used an approximately 240-gene panel to define spatially organized cellular communities critical for morphogenesis.^56^ Higher transcriptome-coverage imaging enables more unbiased discovery at subcellular resolution, but at the cost of significantly longer acquisition times and substantial data storage demands. For most cardiac applications, targeted panels remain the practical choice for validation, although they typically require parallel single-cell or single-nucleus RNA-seq to assign cell identity. Whole-transcriptome spatial platforms, whether sequencing-based (Visium HD) or imaging-based (CosMx whole transcriptome), can in principle support de novo cell typing without a separate reference, though this remains computationally demanding in cardiac tissue.

Technological progress in this field is rapid. 10x Genomics announced Atera in April 2026, an in situ platform offering whole-transcriptome profiling (approximately 18,000 genes) at single-cell sensitivity on both fresh-frozen and FFPE tissue, with higher throughput than current imaging platforms.^57^ Systems of this class further erode the distinctions between sequencing-based discovery and imaging-based validation. No cardiac data have yet been published on this platform.

### A practical decision tree and workflow

For most human heart projects, a hierarchical 2-stage strategy is more realistic than committing to a single “one-shot” platform. In the first stage, relatively low-plex or morphology-focused readouts (eg, H&E or a small immunostain panel) can be used to screen whole sections and select structurally or clinically interesting areas (eg, infarct border zone, conduction system, fibrotic patches). Alternatively, ST (eg, Visium or Visium HD) can be run on representative sections to delineate broad spatial domains (infarct vs remote, viable vs scar) and nominate candidate pathways without strong a priori bias. In the second stage, these preselected regions of interest can then be revisited with higher-resolution or higher-plex methods, such as in situ imaging, high-plex protein or RNA panels, or deeply profiled single-cell and single-nucleus data sets, to validate candidates with cellular precision, refine cell-state definitions, and map cell-cell interactions in context. These reference data sets also serve a practical role in the discovery phase, guiding bin-to-cell aggregation in high-density spatial assays and informing cell-type identification in deconvolution workflows. Hierarchical workflows that first define ROIs on low-plex whole-tissue scans and then apply high-plex imaging or mass cytometry to those ROIs have already been demonstrated in other tissues,^58^ and are naturally extensible to the human heart. That said, this 2-stage paradigm is evolving. Platforms offering both whole-transcriptome coverage and subcellular resolution in a single experiment, such as Visium HD for sequencing-based capture and CosMx whole transcriptome for imaging, are making 1-shot workflows increasingly feasible, particularly as segmentation algorithms and bin-to-cell aggregation tools mature (Figure 3).

**Figure 2:**
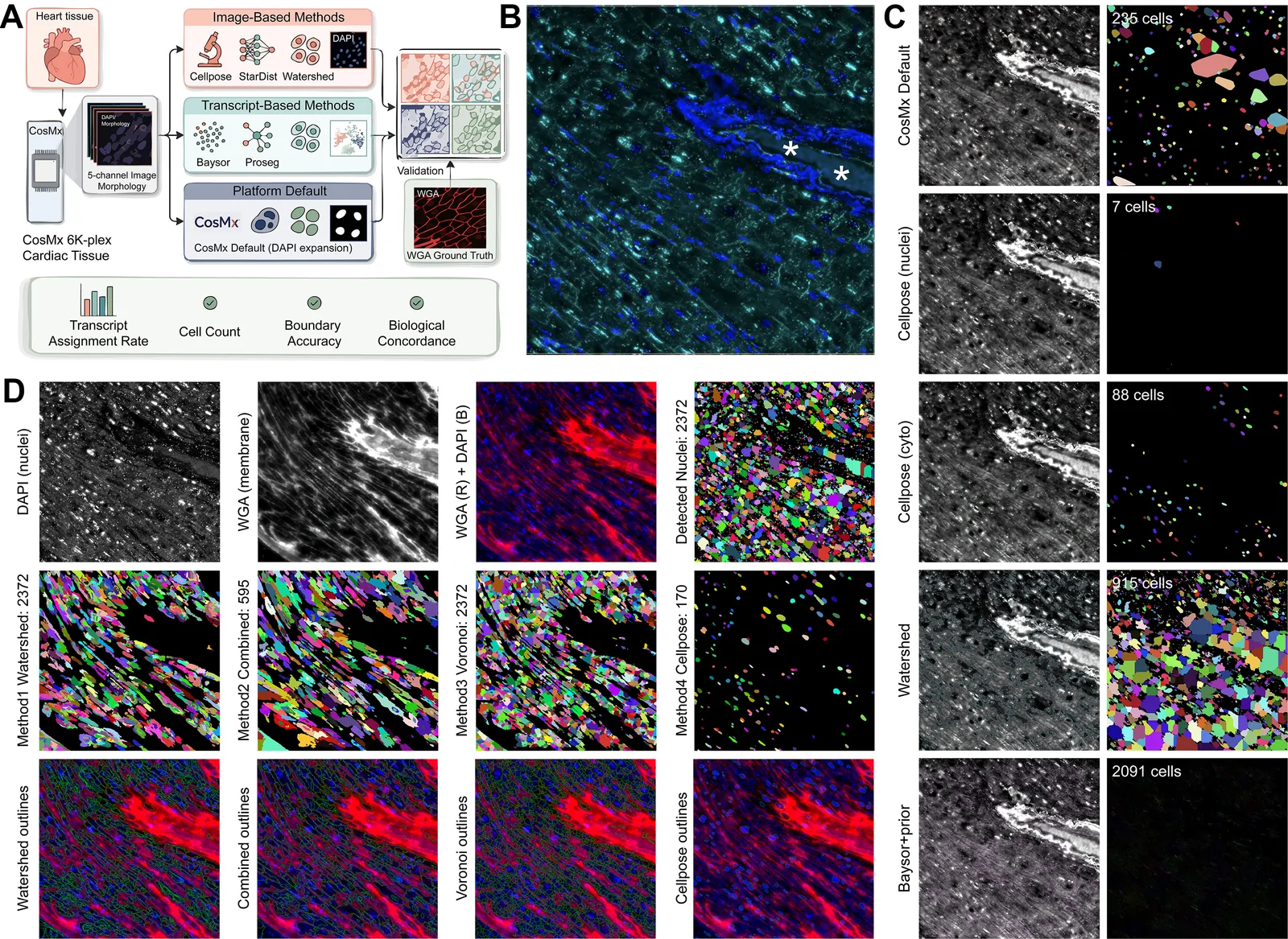
Wheat Germ Agglutinin (WGA)–Based Segmentation Improves Cardiac Cell Boundary Detection (A) Schematic overview of the segmentation benchmarking workflow. (B) Representative field of view (field of view 395) from a human left ventricular section showing CosMx 4′,6-diamidino-2-phenylindole (DAPI) nuclear staining. A transmural blood vessel (asterisks) served as the anatomical landmark for manual registration between the CosMx and confocal coordinate systems. (C) DAPI-only segmentation output across methods for the same field of view. Left column, DAPI reference image; right column, resulting cell masks with detected cell counts. Methods shown top to bottom: CosMx default (235 cells), Cellpose nuclei (7 cells), Cellpose cyto (88 cells), Watershed (915 cells), and Baysor with nuclear prior (2,091 cells). Cell counts vary by more than 2 orders of magnitude across methods: Deep-learning approaches substantially undercount cells in cardiac tissue, whereas transcript-based methods recover implausibly many, consistent with fragmentation of single large cardiomyocytes. (D) Boundary validation using WGA membrane staining registered to CosMx coordinates by manual landmark alignment (scale = 525 px/mm). Top row: DAPI nuclei, WGA membrane signal, merged WGA (red) and DAPI (blue) overlay, and detected nuclei (n = 2,372). Middle row: segmentation masks from Watershed seeded on DAPI nuclei with WGA gradients as boundaries (2,372 cells), combined distance transform (595 cells), Voronoi expansion from nuclear seeds (2,372 cells), and Cellpose with dual-channel input (170 cells). Bottom row: corresponding boundary outlines overlaid on the WGA image. Watershed with DAPI seeds and WGA boundaries produced the closest correspondence to membrane-defined cell borders; Cellpose underperformed on the elongated, irregular cardiomyocyte morphology.

**Figure 3:**
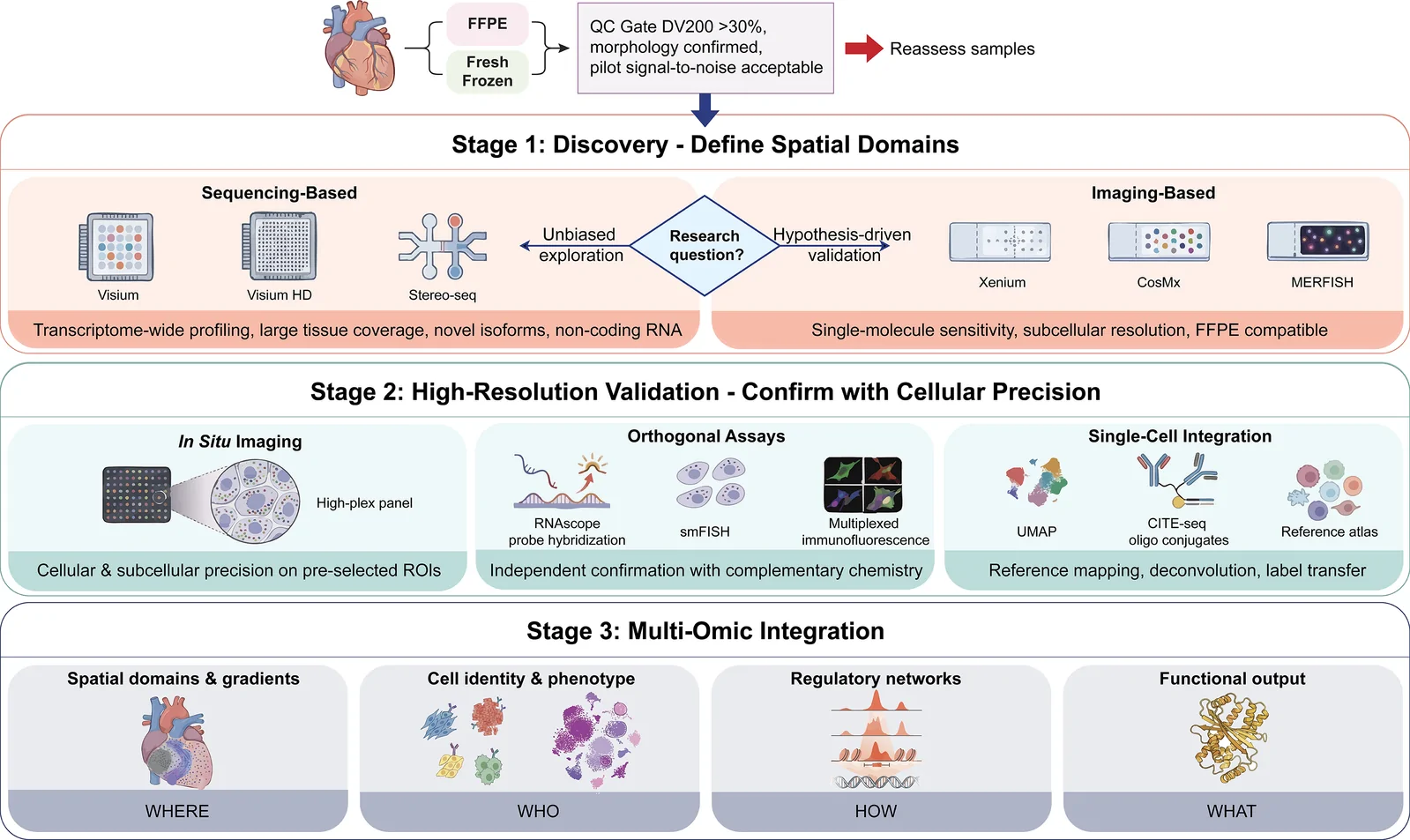
Two-Stage Spatial Transcriptomics Workflow With Multiomic Integration for Human Heart Tissue Fresh-frozen or formalin-fixed paraffin-embedded (FFPE) archived cardiac tissue enters through a quality gate (DV200 > 30%, morphology confirmation). In stage 1 (Discovery), sequencing-based platforms (Visium, Visium HD, Stereo-seq) provide whole-transcriptome coverage for unbiased domain mapping, whereas imaging-based platforms (Xenium, CosMx, MERFISH) offer subcellular resolution for hypothesis-driven validation. Candidate regions and pathways nominated during discovery advance to stage 2 (Validation) through 3 complementary tracks: In situ imaging for cellular precision, orthogonal assays (RNAscope, single-molecule fluorescence in situ hybridization [smFISH]) for independent confirmation, and single-cell integration for reference-based cell identity mapping. Validated candidates enter stage 3 (Multiomic Integration), where 4 modalities each contribute a distinct evidence layer: spatial transcriptomics provides domain coordinates (“where”), snRNA-seq and cellular indexing of transcriptomes and epitopes by sequencing (CITE-seq) resolve cell identity (“who”), scATAC-seq reveals regulatory networks (“how”), and proteomics confirms functional output (“what”). Cardiac tissue preparation considerations are detailed in Figure 1. HD = high definition; MERFISH = multiplexed error-robust fluorescence in situ hybridization; QC = quality control; RNA = ribonucleic acid; ROI = region of interest; UMAP = uniform manifold approximation and projection.

**Central Illustration:**
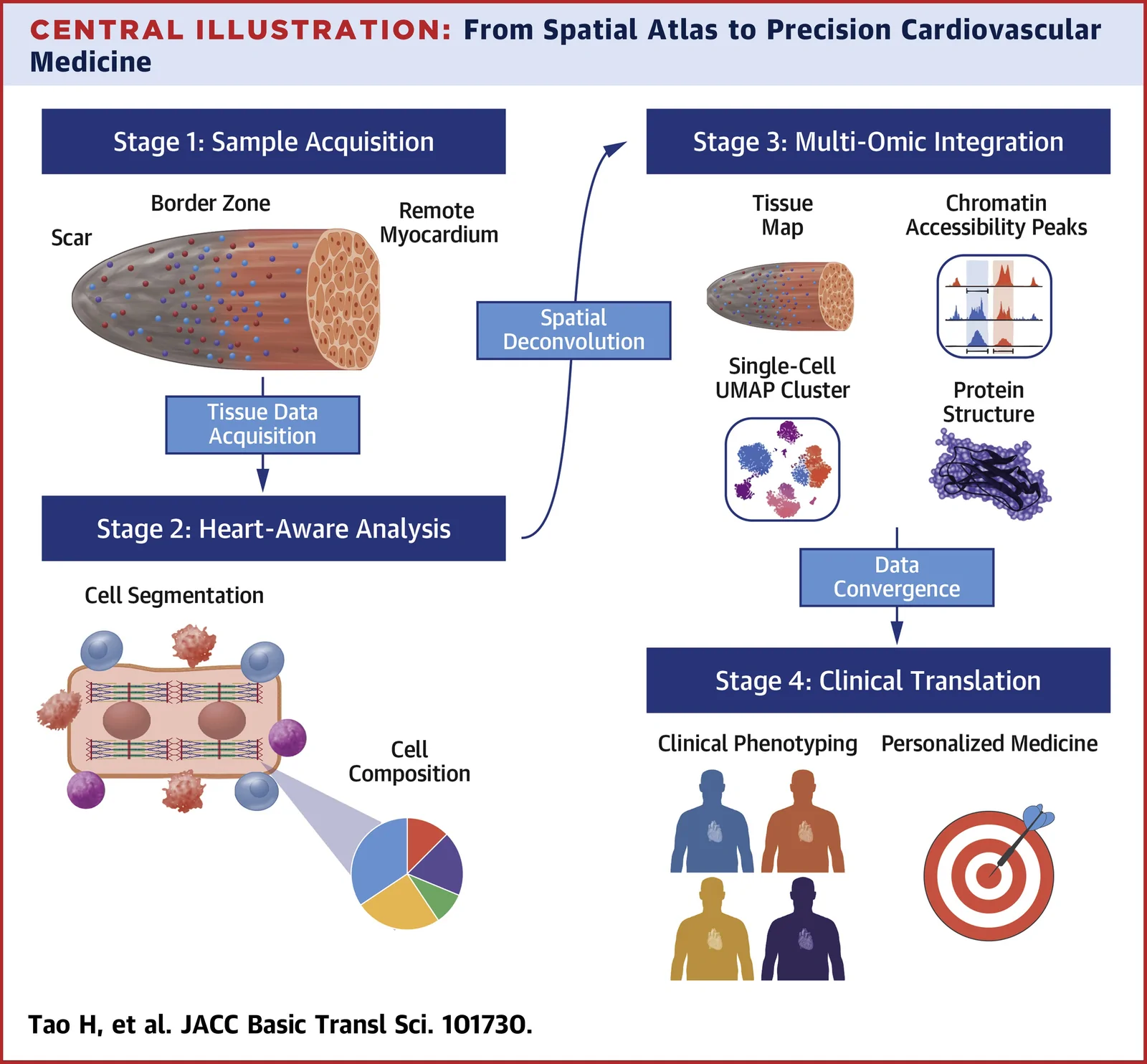
From Spatial Atlas to Precision Cardiovascular Medicine Spatial transcriptomics captures gene expression within intact human cardiac tissue, preserving the architecture of disease zones. Heart-aware analysis resolves cellular composition through optimized segmentation and deconvolution, multiomic integration prioritizes candidate targets, and clinical translation links spatial molecular signatures to clinical phenotyping (eg, cardiac magnetic resonance imaging, computed tomography, echocardiography, positron emission tomography) for precision therapy.

To make these workflows succeed in human heart tissue, it is essential to anticipate and manage cardiac-specific technical pitfalls.^23^^,^^59^ In sequencing-based assays, dense collagen scars often impede permeabilization; if capture is low in fibrotic regions, tissue optimization should be re-run to adjust permeabilization times within safe bounds, while also verifying tissue contact over the capture area. Moreover, for imaging platforms, the strong autofluorescence from lipofuscin and collagen must be mitigated by using platform-compatible quenchers (eg, Sudan Black B), prioritizing far-red detection channels where background noise is lower, and including unstained controls to refine exposure settings.^40^ Furthermore, to minimize segmentation errors in these high-resolution assays, thinner sections (4-6 μm) are preferred to reduce cell overlap, and nuclei vs whole-cell masks should be validated in representative ROIs. In addition, across all workflows using FFPE, over-fragmented RNA represents a silent failure mode; it is critical to track block age, prioritize ROIs with preserved morphology, and consider the DV200 metric on adjacent curls as a quality gate.^60^ Finally, to address the run-time constraints of high-plex imaging, studies should restrict imaging to hypothesis-driven ROIs identified during the discovery phase or tile fewer, larger ROIs with a balanced panel design (Figure 1).

## Heart-Aware Data Analysis for Translational Readouts

For human myocardium, ST analysis has several interconnected goals: Recovering anatomically meaningful domains (eg, infarct, border, perivascular), resolving cell-type composition, quantifying spatial patterns with uncertainty, and producing ranked, reproducible candidates for validation. This workflow demands specific adaptations to handle the heart's cellular architecture.

### Preprocessing and quality control: Beyond standard metrics

Rigorous quality control (QC) is essential. For sequencing-based capture, tissue masks generated from histology exclude off-tissue areas. But standard filtering, such as removing spots below a global transcript count threshold, can unnecessarily discard biologically relevant low-count regions like fibrotic scars. Spatially aware methods such as SpotSweeper^61^ are recommended; unlike standard outlier detection, this algorithm uses local neighborhood comparisons to distinguish true biological heterogeneity from technical artifacts like tissue folds.

For in situ imaging, QC focuses on decoding accuracy and segmentation. Benchmarking of platforms like Xenium reveals that whereas detection sensitivity is high, segmentation remains the primary analytical bottleneck.^54^^,^^62^ Standard nuclear expansion-based algorithms often misassign transcripts in dense or irregular cardiac tissue. Recent evaluations suggest that segmentation-free analyses or deep-learning-based refinement (see Cell Segmentation) can outperform default vendor workflows. Users must also remain critical of “whole transcriptome” imaging claims; as panel size increases, fewer probes can be allocated per gene to avoid optical crowding, reducing per-gene sensitivity. In dense cardiac tissue, this trade-off is compounded, necessitating rigorous, transcript-density-aware QC.^54^ Standard normalization methods (eg, scaling to 10,000 counts) assume cells have similar total RNA content. In the heart, this assumption breaks down because a single cardiomyocyte contains roughly 10-20 times more RNA than a fibroblast or immune cell, reflecting its size and metabolic demands. To correct for this, the ReDeconv method^63^ explicitly models these “transcriptome size” differences using a “Count based on Linearized Transcriptome Size” normalization. This prevents the artificial inflation of immune cell proportions and shrinkage of cardiomyocyte signals, leading to more accurate downstream analysis.

### Cell segmentation: Challenges and cardiac-specific solutions

Segmentation is the single largest source of error in imaging-based spatial analysis. Two distinct families of approaches have emerged, and they rest on different assumptions. Image-based methods infer boundaries from morphology and then assign transcripts to whatever falls within them; they are fast, produce interpretable masks, and fail wherever the membrane is not visible. Transcript-based methods infer boundaries from the spatial statistics of the transcripts themselves; they require no membrane channel and can recover cells that imaging misses, but they depend on adequate transcript density and can impose boundaries where none exist. However, cardiac tissue can strain some of the assumptions inherent to both: cardiomyocyte membranes are poorly resolved by the nuclear and epithelial markers most platforms provide, whereas the transcript density within a single cardiomyocyte is high enough that transcript-based clustering may split 1 cell into several. The practical answer in myocardium is usually a hybrid. We take each family in turn.

#### Image-based segmentation

In most tissues, independent benchmarks recommend generalist deep-learning models over vendor algorithms. Cellpose^28^ and its foundation-model successor Cellpose-SAM^29^ are trained predominantly on cells that are small, round, and mononuclear, and Mesmer,^30^ trained on the TissueNet data set, infers whole-cell boundaries from nuclear staining alone, which is useful where membrane markers are unavailable. Adult cardiomyocytes are none of these things, and in our hands these models perform poorly on myocardium without either fine-tuning or an additional membrane channel to constrain boundaries (Figure 2). Cardiac-specific training is the emerging alternative. CaMVIA-3-dimensional, a volumetric deep-learning pipeline, segments cardiomyocytes in 3-dimensions and has shown that pathogenic-variant-positive hypertrophic cardiomyopathy is characterized by concentric cellular hypertrophy, whereas variant-negative disease is driven predominantly by extracellular volume expansion.^64^ Notably, that work proceeds in the opposite direction to ST: Morphology is measured first, and expression from dissociated single nuclei is mapped onto it afterwards. The spatial transcriptomic problem is the reverse. Transcripts are localized first, and the boundaries needed to assign them must be recovered from imaging that was never optimized for that purpose.

#### Transcript-based segmentation

Even with optimized boundaries, dense tissue produces spatial doublets. Transcript-based refinement algorithms such as FastReseg^65^ score the goodness-of-fit of transcripts within each cell and split segments where the profile suggests a mixture. These approaches form a modular pipeline: initial boundary detection (Voronoi, watershed, or Cellpose), optional membrane-guided refinement, and transcript-based refinement (FastReseg) to resolve residual doublets. Baysor^55^ offers an alternative approach, constructing segmentation directly from gene neighborhood graphs; it can operate independently or incorporate nuclear masks as a prior. In cardiac tissue, the choice matters: without a prior, the transcript density within a single large cardiomyocyte can be sufficient to fragment it into several apparent cells.

#### Segmentation-free approaches

A third strategy bypasses boundary detection entirely. TopACT^66^ classifies cell types directly from transcript point clouds. More recently, several segmentation-free analytical methods have been made available to analyse spatially resolved transcriptomics data. FICTURE infers spatial cell-type patterns by dividing tissue into individual pixels containing some counts of genes, overlaying the pixels with artificial “lattice points” that overlap spatially (thus each pixel can be influenced by several such anchors), and probabilistically assigning cell types to each anchor. Gene expression at each pixel can then be assumed to arise from latent factors associated with its anchors and assigned the appropriate cell type(s).^67^ In comparison, Sainsc is spot-based spatial cell-type analysis-based and calculates the kernel density estimation for each gene. Each pixel can then be represented as a vector of local expression of all genes, approximating the gene expression profile of a hypothetical cell in the region. The computational tool then calculates the cosine similarity of that pixel to known cell-type signatures (typically derived from an existing single-cell RNA-seq data set). Every pixel is assigned a best and second-best score with a probability associated with each.^68^ These methods are attractive in tissues where cell boundaries are difficult to define, though whether they outperform boundary-based methods for recovering large cardiomyocytes remains to be tested.

#### Membrane staining as a cardiac-specific solution

A practical solution for cardiac tissue is supplementary membrane staining with wheat germ agglutinin (WGA), which binds N-acetylglucosamine and sialic acid residues on cell membranes, providing boundary delineation that nuclear-only approaches cannot achieve in cardiomyocytes.^69^^,^^70^ The principle is established in other settings: postrun WGA staining has been used to define anatomical axes following Xenium acquisition in intestine,^71^ and WGA-guided deep learning has been used to reconstruct cardiomyocyte boundaries in 3-dimensions.^64^^,^^72^ It is worth noting that cardiomyocytes form a functional syncytium, connected longitudinally through gap junctions at intercalated discs. WGA delineates these disc boundaries, but whether individual cardiomyocyte segmentation captures biologically meaningful transcriptional units, as opposed to syncytial domains, remains an open question. The default CosMx segmentation relies on a protein panel (PanCK, CD68, CD298/B2M, CD45) designed for epithelial and immune cell identification, markers that provide little membrane contrast for cardiomyocytes.

We illustrate a postacquisition WGA workflow on CosMx (Figure 2): Tissue sections were stained with WGA and imaged on a confocal microscope. The observations below are our own, previously unpublished, and are offered as preliminary workflow experience rather than as a new method; a systematic benchmark against alternative segmentation strategies will be reported separately. Registration to CosMx coordinates proved challenging because of differences in imaging systems and lack of fiducial markers; we achieved acceptable alignment through manual landmark identification using anatomical features (blood vessels) as reference points.

For segmentation, we compared 4 approaches combining 4',6-diamidino-2-phenylindole (DAPI) and WGA: Marker-controlled watershed using DAPI-detected nuclei as seeds and WGA gradients as boundaries, combined distance transform, Voronoi expansion from nuclei, and Cellpose with dual-channel input. Watershed with DAPI + WGA achieved the best boundary detection, whereas Cellpose underperformed, consistent with the training-data mismatch described above. Two practical points deserve emphasis: Cross-platform image registration requires careful landmark selection or, ideally, fiducial markers placed before imaging. Furthermore, accurate alignment is critical, since even small misregistrations propagate into systematic transcript misassignment. The workflow adds 2-4 hours of confocal time per slide.

Although demonstrated here with CosMx, this approach is in principle applicable to any imaging-based platform that preserves the tissue section. Basement membrane markers such as laminin may offer complementary boundary information, although their performance on FFPE cardiac tissue after ST acquisition remains to be evaluated. For smaller interstitial cells such as fibroblasts and macrophages, morphologic segmentation alone cannot reliably distinguish cell types; instead, identity is resolved downstream through transcript-based clustering or label transfer from single-cell references.

A systematic comparison of these strategies will be needed to establish best practices for cardiac tissue. The general principle holds across image-based, transcript-based, and artificial intelligence approaches alike: No algorithm can recover a boundary that was never imaged.

### Cell identity: Deconvolution and label transfer

Once cells or spots are defined, the next step is assigning biological identity. For resolving cell mixtures in spot-based assays (eg, Visium), probabilistic deconvolution frameworks are essential. Methods like RCTD,^73^ cell2location,^74^ and Stereoscope^75^ use scRNA-seq as a reference to estimate cell-type mixes per spot. Comparative studies indicate that these methods perform well across diverse data sets, with selection often depending on computational resources and the need for Bayesian uncertainty estimates. Stereoscope, for instance, is a widely used reference method that models count data directly rather than normalized scores, providing a rigorous statistical basis critical for quantifying subtle shifts in cell composition in border zones.^75^ For imaging-based platforms where individual cells are segmented, label transfer replaces deconvolution: Methods such as Seurat's anchor-based transfer and InSituType project reference atlas labels onto spatially resolved cells, assigning identity based on transcriptomic similarity rather than mixture modeling.

### Multisample reproducibility and integration

Single-sample workflows establish cell identities and spatial domains; scaling to patient cohorts introduces batch effects that must be addressed before cross-sample comparisons. Unlike single-cell RNA-seq, where batch correction is routine via Harmony or reciprocal principal component analysis, ST lacks integration frameworks that simultaneously correct batch effects while preserving spatial coordinates; spatially-aware tools like GraphST^76^ are a first step, but standardized multisample pipelines remain an open challenge. Cross-patient consistency should be quantified using domain overlap metrics (eg, Jaccard indexes), and effect sizes must be replicated across patients to separate shared disease signatures from individual variation. Method concordance adds a further check: Comparing deconvolution proportions across at least 2 independent algorithms guards against tool-specific artefacts.^77^ Deconvolution results should include confidence intervals, cell-state assignments should report probabilities, and sensitivity analyses varying filtering thresholds and reference panels should confirm that conclusions are robust to parameter choices. Minimum reporting should include QC metrics, exact ROI coordinates, and code versions.

### Spatial domains and cellular niches

With batch-corrected, multisample data sets in hand, the analysis turns to defining spatially coherent tissue domains and the cellular neighborhoods within them. Defining spatial domains requires integrating histology with gene expression. Standard unsupervised clustering in Seurat or Scanpy can identify transcriptionally distinct regions, and spatially-aware extensions like GraphST^76^ incorporate tissue coordinates to improve domain coherence. Probabilistic frameworks like Starfysh^78^ explicitly model this relationship, using “spatial hubs” to refine the definition of transitional states (eg, infarct border zones) that purely transcriptomic clustering might blur. Furthermore, recent multimodal studies^79^ have established workflows for defining “inflammatory niches” based on specific cell-cell colocalization patterns. Such niche analysis has recently proven powerful in the heart: integrating scRNA-seq with high-resolution ST revealed a rare population of “progenitor-like cardiomyocytes” whose cell-cycle activity was promoted by signals from a specific myeloid and lymphoid niche, offering new targets for repair.^80^

### Mechanosensing and spatial mechano-transcriptomics

Beyond cell types, functional inference is an emerging concept. The heart is a mechanical organ, yet most ST analysis is purely molecular. Mechanosensing represents a particularly important gap in the cardiac spatial field. Mechanosensitive gene programs are increasingly recognized as active drivers of cardiac remodeling rather than simple secondary consequences of tissue injury. In the fibrotic heart, mechanical cues can directly promote the activation of cardiac stromal cells through Yes-associated protein (YAP)-dependent transactivation, placing mechanosensitive signaling upstream of profibrotic transcriptional programs.^81^ More recently, selective inhibition of stromal mechanosensing was shown to suppress cardiac fibrosis by targeting the fibroblast-enriched mechanosensor SRC kinase., further supporting the view that mechanical signaling actively sustains profibrotic stromal states rather than merely reflecting tissue injury.^82^ More broadly, cardiac fibroblasts are now understood as central integrators of extracellular matrix-derived signals and key orchestrators of intercellular crosstalk across the myocardium, reinforcing the concept that stromal mechanobiology is an active component of cardiac remodeling.^83^ Extending these observations across cardiac cell types, spatial transcriptomic analysis after myocardial infarction (MI) has shown that the infarct border zone contains a distinct cardiomyocyte population enriched for mechano-sensing genes, including *Csrp3*, and that modulation of this program influences postinfarction remodeling.^84^ Together, these findings support the idea that mechano-sensing genes are not only important in cardiac pathology but can also serve as spatially resolved molecular readouts of local mechanical stress within diseased myocardium.

Tools are now emerging to interrogate this relationship more directly. Spatial mechano-transcriptomics provides a framework to combine spatial gene-expression data with cell morphology and tissue architecture to infer local mechanical states at single-cell resolution.^85^ This approach generates spatial maps of inferred mechanical variables, including cell–cell junctional tension, intracellular pressure, and cell stress, and then relates these mechanical features to transcriptional profiles. This makes it possible to identify which regions of a tissue are under higher mechanical load, and which genes are spatially associated with tension or compression independently of simple positional effects. In this context, spatial mechano-transcriptomics may help uncover previously unrecognized mechanosensitive markers and determine how mechanical heterogeneity contributes to spatially restricted disease programs, while also pointing to candidate therapeutic targets in diseased myocardium.

### Multimodal integration

ST gains further power when integrated with orthogonal molecular data. ST provides location; single-cell or single-nucleus RNA-seq provides cell identity; CITE-seq adds surface protein markers; ATAC-seq reveals regulatory accessibility; proteomics captures functional output. These modalities differ in temporal dynamics and technical noise, as RNA changes within hours, proteins persist for days, chromatin accessibility reflects yet another timescale, and so integration requires deliberate study design.^86^

Effective cross-modal integration is best designed from the outset, rather than post hoc alignment. First, context matching is paramount; references should ideally be built from the same anatomical region and disease state (eg, ventricular myocardium in ischemic vs nonischemic conditions) to ensure that cell states mapped spatially actually exist in the tissue, as highlighted in recent single-cell and spatial reviews of heart development and disease.^87^ Second, to prevent overfitting and circular reasoning, training references should remain independent from the ST sections being evaluated (ideally from held-out patients or complementary cohorts) and, where possible, analyses should be repeated with an alternative atlas to test robustness to reference choice. Finally, as emphasized under Multisample Reproducibility, reproducibility must be the standard, summarizing effect sizes and concordance across multiple patients and at least 2 computational methods, while preserving probabilistic uncertainty scores to avoid overconfidence in associations.

The most established workflow couples ST with sc/snRNA-seq to resolve cell identities. Because spot-based ST captures mixtures, it strongly benefits from, and is often interpreted through, high-resolution single-nucleus references to deconvolute these mixtures into cell states.^88^ A prime example is the comprehensive single-nucleus atlas of human dilated and hypertrophic cardiomyopathy.^89^ This study defined the specific transcriptomic signature of “activated fibroblasts” (eg, *POSTN*^*+*^, *FAP*^*+*^) that are unique to failing hearts. Spatial workflows now rely on such curated references to physically locate these rare pathogenic populations within fibrotic niches, validating their spatial segregation from quiescent fibroblasts in remote myocardium. CITE-seq can further refine these references by providing simultaneous transcriptomes and surface proteins. In the human heart, CITE-seq across donor, acute MI, and chronic cardiomyopathy samples has revealed distinct fibroblast and macrophage subsets linked to fibrotic burden, which would be difficult to delineate from RNA alone.^90^ Subsequent mechanistic work validated these findings, demonstrating that activated fibroblasts in the infarct border zone are driven by macrophage-derived IL-1β signaling through a BRD4-dependent MEOX1 transcriptional program, with BRD4 inhibition attenuating fibrosis in vivo.^91^ Importantly, CITE-seq panels are finite and typically enriched for immune markers, so they preferentially sharpen immune cell phenotypes; fibroblast or endothelial states may remain under-resolved unless explicitly targeted in panel design.

Moving beyond cell identity to regulatory mechanisms requires scATAC-seq integration. By linking chromatin accessibility peaks to gene expression, researchers can identify the upstream transcription factors driving spatially restricted cell states. The “Multiomic Map” of human MI^1^ is a key example of this approach. By projecting chromatin accessibility data onto spatial transcriptomic maps, the authors predicted and validated specific enhancers regulating *FN1* and *POSTN* expression in border-zone myofibroblasts, effectively linking an epigenetic state to a structural niche. Similarly, recent work in oncology^20^ combined ST with regulatory network analysis to identify transcription factors driving immune-evasion “hubs,” a strategy directly applicable to identifying regulators of immune exclusion in cardiac scars. However, inferring “causal” regulators from chromatin–expression correlations remains fundamentally model-based: peak-to-gene links, transcription-factor activities, and regulatory networks are all estimated using prior-knowledge databases and motif predictions, so regulatory assignments should be treated as hypotheses for perturbational testing rather than definitive mechanisms.

To provide functional confirmation, proteomic integration is now possible, particularly for extracellular matrix components and secreted cytokines where RNA proxies may not always correlate with protein abundance.^92^^,^^93^ Emerging platforms like DBiT-seq now enable true same-section coprofiling of transcriptomics and proteomics through microfluidic barcoding, eliminating alignment errors inherent in serial-section approaches.^94^ Spatial proteomics via highly multiplexed immunofluorescence or mass-spectrometry-based approaches, allowing researchers to test whether domain-specific RNA programs translate into local protein accumulation, or whether post-transcriptional regulation decouples the 2.^95^ Notably, many extracellular matrix and secreted factors have long half-lives and diffuse across domains, so spatial proteomics may reveal broader or shifted distributions than the underlying RNA; this discrepancy can itself be biologically informative. Finally, even highly ranked candidates must be benchmarked against existing clinical predictors; recent cardio-omics studies emphasize that multiomic signatures only add translational value when they improve upon simpler clinical and imaging markers in independent cohorts.^86^

Ultimately, the goal of multimodal synthesis is evidence triage. Rather than pursuing every spatially variable gene, candidates should be scored and ranked based on a composite of: 1) spatial strength (effect size and autocorrelation), 2) cellular plausibility from sc/snRNA-seq and CITE-seq, 3) regulatory support from scATAC regulons, and 4) protein concordance. This rigorous filtration produces a prioritized list of targets complete with domain masks, effect sizes, and uncertainty metrics that can be confidently handed off for validation via RNAscope or clinical imaging correlation (Figure 3).

### Analytical deliverables

The final output of the computational pipeline must be structured to directly feed biological interpretation. This deliverable should include: 1) a confidence-scored domain map per section, 2) ranked pathway activities and differential expression lists per domain, 3) cell-type compositions with quantified uncertainty, and 4) a prioritized list of ligand–receptor pairs filtered by spatial constraints. These outputs serve as the evidence trail for the subsequent phases of biological validation and clinical integration (Table 2).

**Table 2:** Computational Tools for Cardiac Spatial Transcriptomics

Task	Tool	Key Feature	Reference
Quality control	SpotSweeper	Local neighborhood comparison	^61^
Normalization	ReDeconv (CLTS)	Corrects for cardiomyocyte transcriptome size	^63^
Deconvolution	RCTD	Robust cell type decomposition	^73^
	cell2location	Bayesian spatial mapping	^74^
	Stereoscope	Probabilistic inference	^75^
Segmentation	Cellpose	Deep learning, requires membrane input for cardiomyocytes	^28^
	bin2cell	Morphology-guided bin-to-cell aggregation for Visium HD	^51^
	Mesmer	Nuclear-only whole-cell inference	^30^
	TopACT	Segmentation-free topological cell typing	^66^
	FICTURE	Segmentation-free pixel-level cell-type assignment	^67^
	Sainsc	Segmentation-free KDE-based expression matching	^68^
Refinement	FastReseg	Transcript-based splitting	^65^
	Baysor	Density-based assignment	^55^
Domain detection	Starfysh	Spatial hubs, histology integration	^78^
Functional inference	scMetabolism	Metabolic pathway projection	^96^

CLTS = count based on linearized transcriptome size; KDE = kernel density estimation; RCTD = robust cell type decomposition; other abbriviations as in Table 1.

## Outlook: From Spatial Atlases to Mechanism and Therapy

ST in human cardiovascular tissue is evolving from descriptive atlases toward decision-support for translational studies. Table 3 summarizes the major human cardiac spatial and single-cell studies published to date, organized by disease context. MI, some cardiomyopathies, and heart failure have been comparatively well studied using ST, reflecting both human and mouse tissue availability and clear translational relevance. However, many other important conditions remain to be fully explored, including but not limited to common arrhythmias and conduction system disease, infiltrative cardiomyopathies, congenital heart diseases, and others.

**Table 3:** Spatial and Single Cell Studies of Human Cardiovascular Disease

Disease Context	Key Study	Platform(s)	Principal Finding	References
Normal adult heart	Kanemaru et al^23^	Visium + snRNA-seq + CosMx	Multiomic cardiac niches, including conduction system	^23^
Normal adult heart (multiregion)	Cranley et al^97^	snRNA + snATAC + Xenium + Xenium 5K + Visium HD	Region-specific cell states and a valve fibroblast–macrophage niche; disease-variant enrichment by region	^97^
Developing heart	Farah et al^56^	MERFISH (approximately 240 genes)	Spatially organized cellular communities in morphogenesis	^56^
Developing heart	Lázár et al^26^	Visium + Stereo-seq	Spatiotemporal atlas of the developing human heart	^26^
Myocardial infarction	Kuppe et al^1^	Visium + snRNA-seq + snATAC-seq	Border-zone myofibroblast enhancer regulation (FN1, POSTN)	^1^
Myocardial infarction (acute)	Wünnemann et al^98^	Spatial multiomics	Endocardium as entry route for neutrophil infiltration	^98^
Cardiac repair	Chan et al^80^	scRNA-seq + high-res ST	Progenitor-like cardiomyocytes promoted by a myeloid–lymphoid niche	^80^
Heart failure (general)	Lee et al^24^	Spatial transcriptomics	Spatial transcriptional landscape of human heart failure	^24^
Heart failure/fibrosis	Amrute et al^90^	CITE-seq + spatial	Immune–fibroblast communication; fibroblast/macrophage subsets	^90^
HCM	Wei et al^64^	3-dimensional volumetric imaging + snRNA-seq	Concentric cardiomyocyte hypertrophy in pathogenic-variant-positive HCM; extracellular volume expansion in variant-negative disease	^64^
Ischaemic heart disease	Linna-Kuosmanen et al^25^	Spatial + snRNA-seq	Proinflammatory microvascular changes in right atrial tissue	^25^
HFpEF	Yamada et al^99^	Spatial transcriptomics	Fibroblast clusters driving focal perivascular fibrosis; repressed by Gata4	^99^
Coronary artery disease	Zhu et al^100^	Spatial multiomics	SPP1^+^ senescence-like macrophages linking NO_2_ exposure to CHD	^100^

CHD = coronary heart disease; CITE-seq = cellular indexing of transcriptomes and epitopes by sequencing; CosMx = CosMx Spatial Molecular Imaging; FN1 = fibronectin 1; Gata4 = GATA binding protein 4; HFpEF = heart failure with preserved ejection fraction; HCM = hypertrophic cardiomyopathy; NO₂ = nitrogen dioxide; POSTN = periostin; scRNA-seq = single-cell RNA sequencing; snATAC-seq = single-nucleus assay for transposase-accessible chromatin sequencing; snRNA-seq = single-nucleus RNA sequencing; SPP1 = secreted phosphoprotein 1; ST = spatial transcriptomics.

### Emerging technologies: standardization and causality

As atlases grow, anatomical standardization becomes non-negotiable. The neuroscience community has set a precedent with unified “Common Coordinate Frameworks” that align variable samples to a standard reference.^101^^,^^102^ The cardiac field may also benefit from similarly rigorous anatomical labeling to ensure that a “border zone” defined in 1 patient biologically corresponds to the same region in another, enabling true cross-cohort integration. Beyond observation, the field is advancing toward causal spatial biology. New methodologies like Perturb-FISH^103^ now allow for simultaneous CRISPR-based genetic screening and spatial transcriptomic readout. By knocking out regulators and visualizing the immediate spatial consequences in situ, researchers can dissect the intracellular and intercellular circuitry driving phenotypes like fibrosis or arrhythmia, rather than merely observing their end-states. At present, however, such perturbational approaches are largely restricted to model systems and limited panels of perturbations, so scaling them to human cardiac tissue and clinically relevant gene sets remains an open challenge.

### Foundation models: the era of “visual-omics”

Artificial intelligence is reshaping analysis through foundation models. Tools like OmiCLIP^104^ use contrastive learning to align histology images with transcriptomic data, effectively teaching artificial intelligence to “read” gene expression directly from H&E slides. This democratizes access to spatial insights for retrospective cohorts where only standard pathology exists. However, the “garbage in, garbage out” principle strictly applies; benchmarking shows that prediction accuracy drops precipitously with histologic artifacts, requiring rigorous image QC to prevent “hallucinated” domains.^62^ To address the “black box” nature of these models, next-generation pathway-aware transformers like PAMT^105^ explicitly model the interaction between histologic patches and biological pathways, identifying specific molecular circuits driving survival predictions. Furthermore, context-aware models like Nicheformer,^106^ trained on over 110 million cells, can now predict the spatial niche of a cell solely from its dissociated expression profile, allowing researchers to “spatialize” vast legacy scRNA-seq data sets. Nonetheless, most foundation models have been trained predominantly on noncardiac data sets, so their predictions in human myocardium should be validated against ground-truth ST before being used for mechanistic inference or clinical decision-making.

### Biological interpretation and clinical translation

The ultimate goal is to link spatial molecular signatures to clinical phenotypes and therapeutic targets.

Imaging linkage (“virtual biopsy”): Standard coregistration of ST ROIs to cardiac magnetic resonance imaging (late gadolinium enhancement, T1/T2, extracellular volume) is creating in vivo correlates of spatial programs. New artificial intelligence-enabled frameworks can automate the screening of cardiovascular conditions from cardiovascular magnetic resonance imaging and link specific texture features of fibrosis directly to spatial transcriptomic signatures.^107^ These models will need robust external validation and careful calibration to avoid overfitting to single-center cohorts.

Mechanistic insights: ST is uniquely positioned to resolve cellular plasticity and immune exclusion. High-resolution mapping has demonstrated how spatial context drives “lineage plasticity” in cancer,^108^ a concept directly applicable to cardiomyocyte dedifferentiation or fibroblast activation in the heart. Similarly, defining “exclusion zones” where immune cells are physically barred from tissue regions^109^ could inform strategies to improve reparative cell infiltration in cardiac scars. The same logic applies in the infarcted heart, where the route by which immune cells enter the myocardium, rather than their abundance, may be the more tractable therapeutic target.^98^

Several studies in Table 3 have moved beyond description to nominate therapeutic targets, and they share a common structure. A cell state is found to be spatially confined rather than diffusely distributed; a candidate regulator of that state is identified; and the prediction is then tested by perturbation. Fibroblast clusters in HFpEF^99^ and senescence-like myeloid populations in coronary disease^100^ both follow this pattern, as does the identification of PPARG down-regulation in failing cardiomyocytes, where pharmacologic restoration reversed remodeling.^110^ The spatial component is not incidental in any of these: it is what distinguishes a pathogenic population from its quiescent counterpart elsewhere in the same heart. To prove causality for such spatial hits, the field is moving toward functional validation in engineered heart tissues,^111^ where specific cell–cell interactions found in patient tissue can be recreated and perturbed in vitro. These platforms, whereas still simplified compared with native myocardium, offer a tractable bridge between in situ association and mechanistic intervention.

## Conclusions

ST of the human cardiovascular system has moved from proof-of-concept to practical reality. The workflow we outline (Central Illustration), from cardiac-specific tissue handling through heart-aware segmentation and normalization to multiomic evidence, may help us deliver reproducible, ranked candidates rather than descriptive catalogues. Three developments will shape the next phase. First, spatial perturbation tools such as Perturb-FISH will allow researchers to test, not just observe, the signaling circuits that maintain spatial disease patterning. Second, foundation models trained on paired histology and transcriptomics may make it possible to impute spatial programs from standard sections, opening archival cohorts at a scale that dedicated spatial assays alone cannot reach. Third, coregistration of insights from spatial molecular signatures with patient data and imaging may begin to close the gap between tissue-level biology and clinical phenotypes. The promise of ST will rest on what can be believed after rigorous handling of high-quality data. This is how spatial observation may become trustworthy evidence in cardiovascular medicine.

## Funding Support and Author Disclosures

Dr Lewis reports consulting and/or advisory board roles for Abbott, GSK, and AstraZeneca and research funding from BMS.
